# Supplementary material for: Implementing a Community-Based Initiative to Improve Nutritional Intake among Home-Delivered Meal Recipients
Source: Nutrients. 2022 Feb 23;14(5):944. doi: 10.3390/nu14050944 (PMC8912420; doi:10.3390/nu14050944)
Supplement: Supplementary file 1 [file nutrients-14-00944-s001.zip › nutrients-1558873-supplementary.pdf]

Supplemental Table S1. Nutrition Content Before and After Expanded Meal Plan.

**Before Opting into Expanded Meal Plan**							
		Protein (g)	Na (mg)	Fat (g)	CHO (g)	Calories (kcal)	Potassium
Cold		27	990	23	80	632	1059
Frozen		29	803	18	68	554	1024
Hot		29	803	18	68	554	1024
Mechanical		29	882	16	81	592	1147
Choice Frozen	Mean	25	697	14	44	406	681
	Std. Dev.	4	213	4	4	50	94
After Opting into Expanded Meal Plan							
		Protein (g)	Na (mg)	Fat (g)	CHO (g)	Calories (kcal)	Potassium
Frozen		29	803	18	68	554	1024
Hot		29	803	18	68	554	1024
Kosher		29	803	18	68	554	1024
Mechanical		29	843	17	74	573	1086
Choice Frozen	Mean	29	800	18	67	545	1000
	Std. Dev.	2	67	2	7	43	93

Supplemental Table S2. Correlations between Disability Status, Nutrition Status vis a vis Age and Income.

		Age	Income	ADL Score	IADL Score	NRA Score
Age	Correlation	1	0.18	0.218*	0.376***	−0.064
	p-value		0.142	0.034	<0.001	0.537
Income	Correlation	0.18	1	−0.026	0.05	−0.107
	p-value	0.142		0.84	0.699	0.402
Activities of Daily Living (ADL) Score	Correlation	0.218*	−0.026	1	0.647***	0.19
	p-value	0.034	0.84		<0.001	0.066
Instrumental Activities of Daily Living (IADL) Score	Correlation	0.376***	0.05	0.647***	1	0.293**
	p-value	<0.001	0.699	<0.001		0.004
Nutrition Risk Assessment (NRA) Score	Correlation	−0.064	−0.107	0.19	0.293**	1
	p-value	0.537	0.402	0.066	0.004	

Correlation is significant at 0.05 level *, at 0.01 level **, at 0.001 level ***.

Supplemental Table S3. Nutrition Content of Meals by Age Groups.

	Age Group					
	**60–69**	**70–79**	**80–89**	90–99	100–109	*p*-value
Pre Meal Change Protein (g)	29	29	29	29	29	0.810
Pre Meal Change Na (mg)	809	792	803	800	803	0.601
Pre Meal Change Fat (g)	18	17	18	18	18	0.820
Pre Meal Change CHO (g)	67	67	68	67	68	0.802
Pre Meal Change Calories (kcal)	548	545	554	548	554	0.913
Pre Meal Change Potassium	1006	1005	1024	1006	1024	0.853
Post Meal Change Protein (g)	26	25	24	25	29	0.107
Post Meal Change Na (mg)	760	734	676	722	803	0.185
Post Meal Change Fat (g)	16	16	14	16	18	0.322
Post Meal Change CHO (g)	54	51	50	59	68	0.863
Post Meal Change Calories (kcal)	470	451	431	491	554	0.666
Post Meal Change Potassium	798	737	719	860	1024	0.821
Post-Pre Protein g	−2	−4	−5	−3	0	0.121
Post-Pre Na mg	−49	−56	−127	−78	0	0.243
Post-Pre Fat g	−2	−2	−3	−1	0	0.369
Post-Pre CHO g	−13	−15	−18	−8	0	0.954
Post-Pre Calories kcal	−77	−94	−123	−56	0	0.639
Post-Pre Potassium	−206	−267	−305	−144	0	0.768

Pre meal = nutritional content of original HDM plan at time of enrollment; Post meal = nutritional content of meals at time of study data extraction (December 10, 2021).

Supplemental Table S4. Nutrition Content of Meals by Monthly Income Groups.

	Monthly Income			
	**<2500**	2500–5000	>5000	*p*-value
Pre Meal Change Protein (g)	29	29	19	<0.001***
Pre Meal Change Na (mg)	811	803	574	0.003**
Pre Meal Change Fat (g)	18	18	12	0.006**
Pre Meal Change CHO (g)	68	68	42	<0.001***
Pre Meal Change Calories (kcal)	555	554	360	<0.001***
Pre Meal Change Potassium	1019	1024	649	<0.001***
Post Meal Change Protein (g)	26	28	19	0.204
Post Meal Change Na (mg)	744	794	574	0.716
Post Meal Change Fat (g)	16	16	12	0.244
Post Meal Change CHO (g)	55	59	42	0.626
Post Meal Change Calories (kcal)	477	498	360	0.357
Post Meal Change Potassium	811	929	649	0.821
Post-Pre Protein g	−3	−1	0	0.535
Post-Pre Na mg	−68	−9	0	0.538
Post-Pre Fat g	−2	−1	0	0.918
Post-Pre CHO g	−13	−9	0	0.505
Post-Pre Calories kcal	−78	−56	0	0.655
Post-Pre Potassium	−208	−95	0	0.355

Pre meal = nutritional content of original HDM plan at time of enrollment; Post meal = nutritional content of meals at time of study data extraction Correlation is significant at 0.01 level **, at 0.001 level ***.(December 10, 2021).
